# Strengthening Primary Healthcare in Jordan for Achieving Universal Health Coverage: A Need for Family Health Team Approach

**DOI:** 10.3390/healthcare11222993

**Published:** 2023-11-19

**Authors:** Yousef Khader, Mohannad Al Nsour, Sara Abu Khudair, Randa Saad, Mohammad Rassoul Tarawneh, Faris Lami

**Affiliations:** 1 Department of Public Health, Faculty of Medicine, Jordan University of Science and Technology, Irbid 22110, Jordan; 2 The Eastern Mediterranean Public Health Network, Amman 11195, Jordan; executive.director@emphnet.net (M.A.N.); randaksaad@gmail.com (R.S.); 3 Global Health and Development, Faculty of Social Sciences, Tampere University, 33520 Tampere, Finland; sara.a.khudair@gmail.com; 4 International Academy of Public Health (IAPH), Amman 11195, Jordan; mtarawneh@iaph.org; 5 Department of Family and Community Medicine, College of Medicine, University of Baghdad, Bab Al Muadham, Baghdad 00964, Iraq; farislami@gmail.com

**Keywords:** primary healthcare, reform, family health team

## Abstract

Achieving Universal Health Coverage (UHC) is a strategic objective of the Jordanian government and has been prioritized in its strategies and plans. However, there are several challenges affecting primary healthcare in Jordan and the health system in general that prevent Jordan from achieving UHC. This paper highlights the importance of team-based care in the form of Family Health Teams (FHTs) to realize Jordan’s goal of achieving UHC. FHTs are a team-based approach that brings together diverse professionals to provide a comprehensive, efficient, patient-centered primary care system that meets the changing needs of Jordan’s population and refugees. However, the implementation of FHT may encounter obstacles, including individual, organizational, institutional, and external barriers. To overcome such obstacles, several actions and processes need to be taken, including political commitment and leadership, implementing good governance and policy frameworks, allocating resources and funding, multisectoral collaboration, and engagement of communities and stakeholders. The successful implementation of FHTs requires participation from government officials, parliamentarians, civil society, and influential community, religious, and business leaders. A strategic policy framework, effective oversight, coalition building, regulation, attention to system design, and accountability are also essential. In conclusion, adopting the FHT approach in Jordan’s Primary Healthcare system offers a promising path towards achieving UHC, improving healthcare access, quality, and efficiency while addressing the unique challenges faced by the country’s healthcare system.

## 1. Introduction

Achieving Universal Health Coverage (UHC) is a strategic objective of the Jordanian government and has been prioritized in strategies and plans [1]. Worldwide, the UHC service coverage index has increased from 45/100 in 2000 to 68/100 in 2021. Specifically, in EMR, there has been commendable progress as well, with the UHC service coverage index increasing from 37/100 to 57/100 between 2000 and 2021. Conversely, the UHC service coverage index in Jordan has remained consistently at 65/100 over the same time period, from 2000 to 2021 [2]. This indicator suggests both the strengths and challenges in Jordan’s healthcare system require further examination and potential interventions for improvement. To realize UHC, three objectives must be met: ensuring access to care for everyone—including the poor and patients with the greatest health needs; providing quality healthcare; and removing financial barriers to accessing healthcare [3].

Jordan is an upper-middle-income country located in the Middle East with an estimated population of 11.4 million in 2023, which is projected to increase to about 12.9 million by mid-2030 [4]. Jordan’s crude birth rate has gradually declined from 23.3 births per 1000 population in 2017 to 20.6 births per 1000 population in 2021 [5]. In 2021, the average life expectancy at birth was 73.3 (75.1 for women and 72.3 for men). The infant mortality rate has remained stable at 17 deaths per 1000 live births from 2017 to 2020 and decreased to 9.5 deaths per 1000 live births in 2021. The Gross Domestic Product (GDP) in Jordan was worth USD 47.45 billion in 2022, according to official data from the World Bank. The GDP value of Jordan represents 0.02 percent of the world economy. Jordan’s healthcare system is made up of a combination of the public, private, international, and charity sectors, as well as councils and institutions [1]. The Jordan Ministry of Health (MoH) provides health services to 60% of Jordanians, mostly through the civil insurance program, and all residents can benefit from subsidized MoH healthcare services [6]. Primary healthcare (PHC) is provided through a network of facilities managed by the MoH, including comprehensive health centers providing the broadest range of services and primary health centers and village health centers providing limited services [1]. As of 2022, there were 122 comprehensive health centers, 365 primary health centers, and 184 village health centers in Jordan [7]. For refugees, the United Nations High Commissioner for Refugees (UNHCR), with the support of the Jordanian Ministry of Health, has been providing medical care to Syrian refugees inside camps over the past few years. However, the healthcare needs of the larger population of refugees living outside the camps are not fully met. Until late 2014, the Jordanian MoH provided healthcare free of charge to all Syrian refugees registered with the UNHCR. However, this was a large burden on the healthcare system, and free access to healthcare was withdrawn. Therefore, Syrian refugees living outside camps are now required to pay out of pocket at the same rate as uninsured Jordanians. 

Several challenges affect the PHC and the healthcare system in general, hindering Jordan from achieving UHC. Such challenges include population growth and demographic transition [1], the escalating burden of non-communicable diseases (NCDs) [8,9,10,11,12,13,14], and a strained economic situation [15]. Additionally, as people’s expectations regarding healthcare services are evolving, with patients having increased access to information, greater awareness of their rights, and a stronger desire to participate actively in their healthcare [16], the health system needs to be reoriented toward promotive and preventive models. The situation necessitates health system reform and innovative strategies, models, and interventions. These models and reforms need to be appropriately applied in PHC, as it is the foundation of the healthcare system.

Here, we present a summary of the gaps and challenges affecting PHC in Jordan, necessitating a health system reform, and provide the rationale for shifting towards a Family Health Team (FHT) approach to ensure efficient, equitable, accessible, responsive, quality, and patient-centered healthcare services for all and achieve UHC. FHTs are organizations that consist of a team of primary care physicians, nurse practitioners, dietitians, social workers, and other professionals who work together to provide PHC services [17].

## 2. Gaps and Challenges in Primary Healthcare in Jordan

### 2.1. Governance

The importance of good governance in maintaining resilient health systems cannot be overstated [18]. While the Jordanian government has expressed its commitment to enhancing PHC, certain gaps hinder progress. A review of documents and reports on PHC in Jordan [1,19,20,21,22,23,24] showed that the national health strategies and plans lack evidence-based research, participatory processes, a defined service package, financing mechanisms, monitoring and evaluation frameworks, and a joint review of progress toward objectives. Additionally, the leadership capacities of the PHC Directorate at MoH need strengthening to enhance accountability for coordinating, monitoring, integrating, and implementing national PHC strategies and policies. Mechanisms for feedback from the private sector are inactive, social accountability principles are poorly applied, and multisectoral action at the PHC level is limited. This inadequate accountability is coupled with a deterioration in public confidence in PHC. 

Furthermore, NCDs account for 78% of all mortality in Jordan, with cardiovascular diseases being the leading cause of death since 2009 [25]. The 2019 stepwise survey highlighted a dire rise in the prevalence of NCDs and their risk factors in the nation [8], especially when compared to the previous 2007 stepwise survey [26]. However, there is a lack of an operational multisectoral plan that oversees the integration of NCDs into the PHC [27], leading to inadequate support for NCD prevention, promotion, early detection, capacity building, palliative care, and research. 

### 2.2. Adjustment to Population Health Needs

To effectively address and respond to changing population health needs, it is necessary to regularly gather and analyze health data, use the information appropriately, and continuously assess and monitor evolving contexts and priorities [18]. However, in Jordan, national health surveys are not conducted periodically—for example, the most recent stepwise survey was conducted in 2019 [8], 12 years after the previous survey in 2007 [26], coupled with insufficient actions implemented based on the survey results. Additionally, routine monitoring and evaluation of population health status are limited, and the absence of population-based national registries to compile data on NCDs (except cancer) [27] further hinders efforts to address these evolving needs.

### 2.3. Financing

PHC in Jordan is underfunded and not prioritized by the government or donors, with significantly lower investment compared to secondary care. In 2015, health spending on PHC was 37% of government health expenditure [22]. The focus of healthcare services is mostly on curative services, with limited financial support provided for health promotion, prevention, screening and early detection, palliative care, or research [27]. Additionally, significant disparities exist in out-of-pocket expenses among different socioeconomic groups, geographic locations, and urban and rural areas [28,29,30].

### 2.4. Physical Infrastructure

While PHC facilities are physically accessible to all [30], some buildings are rented, old, cannot be expanded, and need renovation, especially the primary health centers and village health centers [31]. The availability and maintenance of equipment also pose problems [29]. Some clinics receive project-related materials and equipment but experience high staff turnover, with no sustainable plan for regular training on equipment usage. 

### 2.5. Health Workforce

Several reports revealed gaps in the management of human health resources in Jordan [1,20,21,22,23,24,30]. Most of the current human resource management practices do not facilitate or support workers’ performance and motivation [30]. The majority of PHC facilities are managed by general practitioners with no vocational training or experience in creating family practice-oriented healthcare teams [30]. Although PHC service providers are eligible for continuous professional development, most training is donor-dependent [30]. There are a limited number of inclusive degrees in primary care and family medicine, and the available degrees focus only on physicians, excluding key providers such as nurses and midwives. Also, available degrees have selective criteria; e.g., one of the promising diplomas is the regional family medicine diploma, which is only open to general practitioners with at least five years of experience after earning their medical degree [32]. Lack of or inadequate training is one of the most important challenges facing the delivery of high-quality services [30]. Serious knowledge gaps are evident in the health workforce in Jordan in general and within MoH in particular, affecting the different aspects of health workforce resource management, as reliable and updated information is crucial for health workforce planning, recruitment, deployment, and retention.

There are discrepancies and variations in the numbers and types of the health workforce available at different health centers, which are not proportional to the catchment area nor the number of registered patients [33,34]. Although the problem of staff shortages in some facilities cannot be ignored [35], the workload is also an issue mainly affected by a combination of factors, including a lack of an appointment system, a lack of staffing norms, high turnover, limited or reduced working hours, and frequent absenteeism [35]. Although it is claimed that community workers support health centers in Jordan, only a few health centers have such workers. There is no guidance on staff responsibility, and quality management is not widely practiced, with no auditing performed and a lack of supportive supervision and tools for supervision. 

### 2.6. Access and Coverage

According to the 2017–2018 Jordan Household Income and Expenditure Survey, 67% of individuals are insured [36]. Individuals are more likely to be insured in rural areas than in urban areas (85% compared to 65%). At public healthcare facilities, all medicines are provided free of charge for patients with health insurance. For those with private insurance, medical coverage (services included) and the level of copayment (i.e., out-of-pocket payment) depend on the specific insurance policy that has been purchased. Non-Jordanians and Jordanians with no health insurance must pay for their medications or seek medications from the UNRWA, UNHCR, or charitable organizations [37]. Financial barriers to primary healthcare, especially among refugees and uninsured people, are still a significant barrier. The National Health Strategy called attention to the underutilization of PHC services throughout the country as well as challenges to equitable access to care. Expanding coverage of primary healthcare services should be a priority to improve access and equity. 

### 2.7. Health Information System

Jordan’s health information system (HIS) collects data from different sources, including MoH health centers [38]. HIS challenges include the absence of a HIS national steering committee [39], insufficient material resources, inadequate human resources trained and qualified to use computerized systems, challenges in identifying required information and performance indicators on a national level, lack of use of unified terms and abbreviations, paper-based data collection, delays in sending required data from healthcare institutions to the central authority, vertical programs with different surveillance requirements and systems which affects data quality, lack of routine quality assurance mechanisms [38], inadequate application maintenance, outdated rules and regulations for electronic information management, and insufficient utilization of data [38,40].

### 2.8. Models of Care

Generally, there is a lack of an effective referral system in Jordan, and bypassing the PHC is common. Gaps in the existing structure include the absence of an electronic system to manage and verify transfers, the absence of communication between general practitioners in PHC centers and the center or hospital to which the patient is referred, and long process times and financial concerns linked with referrals. There is also misuse of referrals, whereby sometimes patients themselves request the referrals, placing an additional and avoidable burden on hospitals as well as extra operational costs. This misuse may be related to lower confidence in the quality of care provided in PHCs [30]. Additionally, there is neither a well-defined nor an endorsed priority benefit package, and the services provided at PHC are not comprehensive. For instance, systems for the early detection of diabetes and hypertension are not in place. Similarly, early detection of prevalent cancers is not provided at the PHC level, nor is palliative care for NCDs. Although counseling, health education, and lifestyle modifications are provided through some initiatives, they are not institutionalized within the system. Moreover, systems for improving the quality of care are almost lacking. NCDs’ community outreach services and home visits are not in operation, and regular training in NCD management and coaching mechanisms for healthcare workers are lacking.

## 3. The Need for Primary Healthcare Reform in Jordan

Improving PHC in Jordan is a crucial step towards achieving UHC. Previous reports have highlighted the need for an efficient health system, an increase in workforce capacity, and improved responsiveness in PHC. The health workforce should be capable of adapting to changing population needs and the growing demand for healthcare caused by demographic, epidemiological, economic, social, and political changes [41]. Strategic investments in the health system and its workforce can bring health benefits to people, contribute to employment and economic growth, and reduce inequalities [42]. To improve healthcare efficiency and save resources, it is crucial to provide early and appropriate treatment for chronic conditions through outpatient care at the PHC level. This can help reduce unnecessary and avoidable hospitalizations [43]. 

Additionally, to enhance the technical efficiency of PHC, new mechanisms for distributing human resources and developing workforce capacity are necessary. With advances in technology, it is crucial to update PHC professional education to equip primary care providers with the necessary skills to effectively utilize technology in healthcare delivery. Soft skills, such as behavior counseling, shared communication, collaboration, and partnership, are also essential for healthcare personnel. Training programs should cover various areas to enable task sharing and interprofessional collaboration, particularly for managing chronic diseases and their risk factors. These require long-term support from both the care team and patients, making a team of providers essential for long-term commitment to patients.

To enhance the efficiency of PHC, it is necessary to use community-based teams efficiently. Nurses and other health professionals can assume new supportive roles in PHC that can reduce the workload of primary care physicians. This approach has the advantage of not having to “reinvent the wheel” because nurses and midwives are used to providing services and have the necessary soft skills. 

Reorganizing Jordan’s PHC system based on teams and connected networks would result in better coordinated and comprehensive care, improved health outcomes, and long-term cost-effectiveness. By adopting person-centered primary care models, teams and networks are expected to provide a range of multidisciplinary services and effectively meet the health needs of the population. Team-based care is a crucial solution to address the high burden of chronic diseases, as over 40% of individuals have multiple chronic conditions [14]. 

Here, we propose an integrated model for PHC that includes multidisciplinary or interprofessional practices with different PHC professionals to reform the healthcare system in Jordan [44,45,46]. This model aims to offer a range of community health services, such as disease prevention and health promotion, curative services, rehabilitation, and chronic disease management, with patient engagement in shared decision-making. Additionally, the model prioritizes patient-centered services. 

## 4. The Rationale for the Family Health Team Approach

The FHT model differs from other models of service delivery in several ways. In traditional solo or group practices, individual physicians or small groups of healthcare providers often operate independently, and patients may see different providers for different aspects of their care. On the other hand, FHTs are designed to offer a team-based approach, with various healthcare professionals working collaboratively to provide comprehensive care to patients. Community Health Centers which are often focused on serving underserved and vulnerable populations, offer a range of healthcare services. FHTs may have similarities to Community Health Centers in terms of offering a wide range of services, but FHTs are not limited to serving underserved populations and can be found in various communities. Another model of care is Comprehensive Care Assisted by technologies, which provide greater accessibility, especially for remote or homebound patients, as care can be delivered from a distance using technology.

The FHT approach has become necessary to meet the healthcare needs of Jordanians and vulnerable populations. This approach helps reduce inefficiencies in healthcare costs while improving access to comprehensive and patient-centered PHC. FHTs are organizations that consist of a team of primary care physicians, nurse practitioners, dietitians, social workers, and other professionals who work together to provide PHC services [17]. Each team is tailored to local health and community needs. It has been established that “FHTs should become the norm for primary care”. The primary investment outcome of an FHT approach is improving access and utilization of a comprehensive package of PHC services based on holistic care of the entire family, emphasizing long-term provider-patient relationships, and improving the quality and efficiency of services. 

A team approach in PHC has shown many benefits. Studies have found that this approach can lead to improved outcomes and performance [47,48], such as lower healthcare costs due to fewer hospitalizations [49]. Patients also report higher satisfaction with their care and quality of life [49]. Continuity of care is also ensured [50]. By using human healthcare resources more efficiently, this approach can reduce health inequalities [51] and improve accessibility [52].

By assembling a team of healthcare professionals, including doctors, nurses, social workers, and other specialists, FHTs are able to offer a comprehensive range of services that address both physical and mental health needs. This approach takes into account social and environmental factors that may be impacting a patient’s health, resulting in a more holistic view of the patient. FHTs provide a multi-faceted platform to tackle cross-cutting issues like diet, physical activity, education, gender-based violence, child protection, poverty, and community development. These teams promote continuity of care, ensuring that patients have a consistent and ongoing relationship with their healthcare providers, which can prevent gaps in care and improve health outcomes over time. By focusing on prevention and early intervention, FHTs can also help reduce healthcare costs and improve the overall health of the population. 

The government of Jordan is fully dedicated to achieving UHC to provide social and financial protection for all Jordanians and refugees. They have prioritized UHC in most health and health-related national strategies and plans [1]. The integration of PHC services with public health through FHT improves population health, decreases healthcare expenditure, enhances the healthcare system’s performance, and ensures equitable access for everyone [53]. Healthier populations [54] develop more resilient and socioeconomically viable communities [55], which increase the resources available for future services, including healthcare for all. FHT is a core component in realizing the ambitions of UHC as a sustainable development goal [56].

The implementation of FHT in Jordan aligns with many recent efforts, initiatives, policies, strategies, and plans in the country. The Jordan 2025 Vision, launched in 2015, is a long-term national strategy with over 400 policies and goals, including health-related targets, that will be implemented through participatory approaches between the government, the business sector, and civil society [57]. Jordan was also one of the signatories of the Salalah Declaration in 2018, reaffirming its commitment to achieving UHC through building equitable, resilient, and sustainable health systems [58]. Under its National Health Strategy (2015–2019), Jordan’s MoH aimed to modernize its PHC system by adopting a family medicine model based on principles of equity, poverty reduction, and the rational use of limited resources [59].

## 5. Barriers and Facilitators for FHT Implementation

The introduction of team-based care is expected to be accompanied by changes in organizational structure and workflow, all of which can present barriers to adoption if not identified early on. Here are some potential obstacles, along with suggested solutions and strategies to help facilitate the adoption process:

### 5.1. Organizational-Level Barriers and Facilitators

Barriers to the successful implementation of FHTs in Jordan include a lack of supportive regulations, limited experience with the FHT model, inadequate infrastructure, fragmented HIS, inadequate continuing education programs on team-based care, and weak primary care information technology infrastructure. There may also be uncertainty regarding individual roles and responsibilities, both for team members and patients. 

To ensure a successful implementation, policymakers need to define clear roles, responsibilities, and scopes of work for FHT team members. The culture of teamwork and necessary skills for effective collaboration need to be developed, as individualism in decision-making is widespread in Jordan, particularly among physicians. Effective management is crucial for the success of FHTs, and poor management practices can hinder their implementation. Suboptimal preparation before implementing FHTs, such as inadequate staff preparation or insufficient strategic planning for resource allocation, can impede FHT adoption. It is essential to provide practical and theoretical training on team-based care and primary care to prepare healthcare professionals for FHT implementation.

### 5.2. Individual-Level Barriers and Facilitators

When adopting the FHT model, healthcare providers may face obstacles due to a lack of motivation and the demands of additional effort. Jordan’s healthcare system has a biomedical approach that focuses on the physical aspects of diseases, which could complicate the transition. To address this, healthcare providers need adequate training to recognize the importance of integrating medical and non-medical care, a core principle of the FHT model.

Patients may also have concerns about FHT, including the reluctance to share personal information with an additional person in the room. To ensure patients are more involved in their care and understand the value of FHT, public education and promotion of the model are necessary. 

## 6. Necessary Actions and Processes for Implementing FHT

To successfully adopt FHT, shifting from a traditional physician-dependent approach to a more team-based one is crucial. In Jordan, several key actions and processes need to be implemented to support the adoption of team-based care. These include political commitment and leadership, creating enabling governance and policy frameworks, rational funding and allocation of resources, multisectoral collaboration, engaging with communities and other stakeholders, and addressing the complex health and social needs of vulnerable patients.

### 6.1. Political Commitment and Leadership

The government of Jordan is highly committed to allocating resources to implement FHT to achieve UHC. This commitment was reflected in addressing UHC as one of the top national priorities in most health and health-related strategies and plans in Jordan. Moreover, the National Health Strategy aims to modernize its PHC system by adopting a FHT approach. To achieve political ground for implementing the FHT, the support and participation of government officials, parliamentarians, civil society, and influential community, religious, and business leaders is required [60]. The FHT is made up of three components that are interrelated and require an enabling policy environment. Firstly, integrated health services necessitate political commitment and leadership to reallocate resources and align systems. Secondly, multisectoral policies require partnerships among various sectors and the involvement of public and private stakeholders, ideally facilitated by political commitment. Thirdly, empowering individuals and communities requires redistributing power to patients so they can take a leadership role in their health. This requires a political foundation and a commitment to the community. To enable the implementation of FHTs in PHC in Jordan, suggested actions include recruiting advocates or champions for FHT in all influential sectors of society, developing a comprehensive vision of FHT, embedding commitment to FHT as a priority across all sectors through policies, communicating extensively about the commitment to improving PHC through FHT, and holding those responsible for implementing FHT accountable.

### 6.2. Governance and Policy Frameworks

Governance requires a strategic policy framework, effective oversight, coalition building, regulation, attention to system design, and accountability [60]. The MoH has a vital role in this process, acting as a steward to engage both health and non-health stakeholders in the implementation of FHT. A policy framework with specific elements and components is essential to achieving high-quality, team-based standards and continuous improvement of services.

MoH’s stewardship role in FHT requires a shift from its traditional provider role to one that focuses on facilitating partnerships, monitoring, regulation, and oversight across multiple sectors. To achieve this, the MoH’s leadership and technical capacity should be strengthened through training and mentoring. It is important to legitimize community involvement in FHT governance and create an organizational culture that supports monitoring and evaluation. Additionally, creating processes for community and civil society participation in FHT implementation in a nondiscriminatory manner, such as having community-elected representatives in governance structures or community advisory councils, is crucial. 

### 6.3. Funding and Allocation of Resources

Compared to other countries, Jordan has lagged in investing in PHC and allocating sufficient funds toward the health sector [19]. Therefore, it is important to update the health financing strategy by collaborating with various stakeholders from both within and outside the health sector, including the ministries of finance. Proper allocation of funds towards infrastructure, educational programs, training, and appropriate technology for healthcare teams is crucial.

### 6.4. Multisectoral Collaboration

To encourage reform and multisectoral collaboration, it is important to educate and train leaders in all sectors on the benefits of engaging with diverse sectors to implement FHT in PHC. This involves creating an internal culture that values collaboration and developing a vision specifically for FHTs to provide direction. Early identification of stakeholders through stakeholder mapping activities is critical for planning multisectoral collaboration activities. It is also important to clearly outline the rationale for multisector collaboration and build trust among stakeholders and sectors to allow for the exploration of ideas and opportunities. Convening an expert group to identify common problems and develop solutions is recommended. Translating stakeholder engagement into an action plan is essential, and maintaining strong leadership to mobilize diverse stakeholders’ inputs and manage relationships is also important.

Continuous sharing of the FHT implementation process with stakeholders, mobilizing funds and resources for partnership activities, and supporting multisectoral technical working group meetings are also necessary. 

### 6.5. Engagement of Communities and Other Stakeholders

There are multiple reasons why involving stakeholders and the community in FHT implementation is important. Firstly, FHTs require intersectoral collaboration, making the involvement of stakeholders from different disciplines crucial for success. Secondly, FHT must be inclusive, ensuring that vulnerable, marginalized, and disadvantaged groups are represented at all levels. Lastly, sustained stakeholder and community engagement is expected to foster a culture of accountability and quality, encouraging the institutionalization of FHTs.

To establish stakeholder partnerships in FHT, several general steps can be taken. These include mapping out stakeholders at early stages, establishing a governance structure for the partnership, providing capacity building and training for stakeholders, and conducting a collaborative quality assessment of implementing the FHT model. To ensure community engagement during the planning and implementation of FHT, it is recommended to follow the East and Downtown East Ontario Health Teams (OHT) Community Engagement Framework [57]. This framework provides a consistent approach to community engagement, guiding the timing and methods of engagement activities as well as the steps and processes to be considered [61].

## 7. FHTs Development

For the development of FHT in Jordan, the following strategies are recommended:

1. Each health directorate has to form an FHT implementation committee to plan and develop FHTs in health facilities within the directorate;
2. The FHT implementation committee will work with the facility manager to develop a customized plan for implementing FHTs in that facility;
3. The FHT implementation committee needs to use a flexible implementation model. Implementing FHTs using a flexible model can help accommodate differences in geographical areas and health centers. A flexible implementation model can be adapted to the size of the health center and the availability of resources and customized to meet the specific needs of different communities. The composition of the FHT needs to be adapted to the specific needs of the health center and the population served. In comprehensive health centers, FHTs may be composed of physicians, midwives, nurses, pharmacists, psychologists, and dieticians. At the village health clinics, FHTs may include full- or part-time physicians, nurses, and community health workers. The MoH has to coordinate with each health directorate, consider redistribution of human resources between geographic areas and health centers, and hire new employees such as psychologists and dieticians. However, this does not mean that each center has to include the expanded FHT. Some comprehensive centers in a geographical area can be designated to include the expanded FHTs who provide specific services for patients in the catchment areas and to patients referred from other health centers. In this case, patients in need of special services (such as mental health services) can be referred to these centers. Another care delivery option is that some members of FHT in one health center can work part-time and cover other health centers in the health directorate according to need. The head and the members of FHT in comprehensive health centers will also be responsible for supervising medical care as well as improving the knowledge and skills of the teams at the primary healthcare centers and village health centers;
4. Each of the team members is reassigned roles depending on the existing task distribution, healthcare worker capacity, and service provision gaps. Training healthcare workers to perform more than one task allows tasks to be shared amongst the team. This allows the team more flexibility when delivering services and frees up more time for physicians and nurses to focus on patient management;
5. The scope of practice for each member of the FHT can be customized to meet the specific needs of the health center and the population served. The scope of practice for FHTs may differ between district and sub-district levels. FHTs in comprehensive primary health centers may focus on providing comprehensive primary healthcare services to a larger population, including services such as family planning, maternal and child health, and chronic disease management. In village health centers, FHTs may focus on providing primary healthcare services to rural populations, including services such as preventative care, acute care, and chronic disease management. This can include expanded roles for certain healthcare professionals or additional training in specific areas of care. For example, a nurse in a health center might be trained to provide psychosocial support services to patients or to provide outreach services to the community. FHTs may engage in different activities depending on their level of implementation. At the district level, FHTs may engage in activities such as establishing referral networks, coordinating with other healthcare facilities, and participating in quality improvement activities. At the sub-district level, FHTs may engage in activities such as conducting community outreach, conducting home visits, and providing basic healthcare services in the community;
6. All members of the team play an integral role in providing care for each patient. In a team-based care model, lay and mid-level healthcare professionals are trained to take on expanded responsibilities. For instance, this could include safely performing clinical tasks and procedures that could otherwise be restricted to higher-level healthcare workers or physicians, such as measuring blood pressure or initiating treatment;
7. Providing training and support to healthcare professionals in the health center can help them understand the role of FHTs and how they can work together to provide comprehensive care to patients. Training can include topics such as team building, communication skills, and patient-centered care;
8. A conceptual framework for regular monitoring and evaluation of FHT should be developed and centered on the effects of its implementation on the organization, provider, and the way they deliver care and interact with patients, as well as patient experience and outcomes. A set of evaluation indicators and metrics needs to be developed to assess different dimensions such as population orientation of PHC, access, comprehensive person care, patient-centeredness, continuity of care, information technology support, appropriateness, acceptability/patient participation, effectiveness, and safety. Throughout the evaluation, FHT structural and organizational characteristics, performance, and patient experience should be examined and assessed across specified primary healthcare domains.

## 8. Conclusions

To realize Jordan’s goal of achieving the UHC, it is crucial to enhance and modernize PHC services through the implementation of the FHT approach. This approach aims to offer a comprehensive, efficient, patient-centric primary care system that caters to the changing needs of the Jordanian people and refugees. However, the implementation of FHT may encounter obstacles, including individual, organizational, institutional, and external barriers. To overcome such obstacles, several actions and processes need to be taken, including political commitment and leadership, implementing good governance and policy frameworks, allocating resources and funding, multisectoral collaboration, and engagement of communities and stakeholders. The successful implementation of FHTs requires participation from government officials, parliamentarians, civil society, and influential community, religious, and business leaders. A strategic policy framework, effective oversight, coalition building, regulation, attention to system design, and accountability are also essential. The same recommendations are pertinent for countries encountering health system gaps analogous to those witnessed in the Jordanian healthcare system.

## Data Availability

Data are contained within the article.
